# A Molecular and Morphological Reassessment of *Diademaceae*

**DOI:** 10.1155/2014/675348

**Published:** 2014-01-12

**Authors:** Hiran A. Ariyawansa, Rungtiwa Phookamsak, Saowaluck Tibpromma, Ji-Chuan Kang, Kevin D. Hyde

**Affiliations:** ^1^The Engineering and Research Center for Southwest Bio-Pharmaceutical Resources of National Education Ministry of China, Guizhou University, Guiyang, Guizhou 550025, China; ^2^School of Science, Mae Fah Luang University, Chiang Rai 57100, Thailand; ^3^Institute of Excellence in Fungal Research, Mae Fah Luang University, Chiang Rai 57100, Thailand

## Abstract

We revisit the family *Diademaceae* based on available sequence data and morphology. *Diademaceae* is characterized by ascomata opening with a flat circular lid and fissitunicate, short orbicular frequently cylindrical, pedicellate asci. Ascospores are frequently circular in section but narrowing to one end with three or more transverse septa, without longitudinal septa, and mostly with a thick sheath. In recent treatments *Clathrospora*, *Comoclathris*, *Diadema*, *Diademosa*, and *Graphyllium* were placed in the family. Following molecular and morphological study, *Clathrospora*, *Comoclathris*, and *Diademosa*, are excluded from the family and referred to *Pleosporaceae*. *Graphyllium* is excluded from *Diademaceae*, based on hysterothecium-like ascomata with a longitudinal opening, and tentatively placed in *Hysteriaceae* with uncertainty; species with hysterothecia have now been accommodated in at least five families. The study accepts only *Diadema* in the family. The status of *Diademaceae* as a distinct family, based on the ascomata opening by a flat circular lid, is thought to be doubtful. Fresh collections of *Diadema* are needed for epitypification and to obtain sequence data to establish if this is a well-resolved family.

## 1. Introduction

Based on the ascomata opening by a flat circular lid, Shoemaker and Babcock [1] introduced *Diademaceae*, which they considered to be a unique family in the order *Pleosporales*. Initially five genera, that is, *Clathrospora*, *Comoclathris*, *Diadema*, *Diademosa*, and *Macrospora*, were included in the family [1]. Other than the ascomata opening by a flat circular lid, the family was characterized by bitunicate and fissitunicate, clavate or ellipsoidal, short pedicellate asci, and applanate or rarely cylindrical ascospores with three or more transverse septa with or without longitudinal septa and usually with a thick sheath and frequently circular in section but narrowing to one end [1, 2].

Species of the order *Pleosporales *with applanate ascospores can be found in three families (*Diademaceae*, *Hysteriaceae*, and *Pleosporaceae*), which differ in the way the ascomata open [1]. Ascomata openings by a flat circular lid are characteristic of *Diademaceae*. In species of *Hysteriaceae* ascomata open via a long narrow slit and species of *Pleosporaceae* open by a central pore [1]. Various authors have included and excluded different genera in* Diademaceae *by giving priority to different morphological characters [1, 3, 4].* Platyspora* had been referred to this family by various authors [4] or was considered a synonym of *Graphyllium* [2, 4] or *Comoclathris* [5]. Lumbsch and Huhndorf [6] assigned *Macrospora *to *Pleosporaceae*, as the genus was considered to be a synonym of *Pyrenophora* and this treatment was followed by Zhang et al. [3, 4]. In the same study, Lumbsch and Huhndorf [6] had also referred *Graphyllium *to *Diademaceae*. Sequence data is now available for some of these genera thus the importance of their morphological characters and overall relationships can now be tested based on molecular phylogeny.

We have been studying the families of Pleosporales in order to provide a natural classification via morphological characterization together with molecular analysis [2–4, 7]. The family *Diademaceae* has been poorly studied and presently comprises five genera [6], but this has not changed since the family was introduced by Shoemaker and Babcock [1]. Given the considerable taxonomic confusion we revisited this family based on phylogenetic analyses of rDNA sequence data coupled with morphological characters. The aims of the study are to (i) discuss the familial placement of the genera in* Diademaceae* and assess whether they represent natural groups, (ii) determine which morphological characters are useful for generic delineation by observing the type species of each genera, and (iii) illustrate the genera to stimulate fresh collections being made so that molecular data can be used to resolve the systematic relationships of the family.

## 2. Materials and Methods

### 2.1. Specimen Examination

The basic methodology used in this study was the same as Ariyawansa et al. [7]. The type specimens were loaned from the US National Fungus Collections (BPI), Agriculture and Agri-Food Canada (DAOM), and New York Botanical Garden (NY). Ascomata were rehydrated in 5% KOH prior to examination and sectioning. Hand sections of the fruiting structures were mounted in water for microscopic studies and photomicrography. The fungus was examined in a Nikon ECLIPSE 80i compound microscope and photographed by a Cannon 450D digital camera fitted to the microscope. Measurements were made with the Tarosoft (R) Image Frame Work program and images used for figures were processed with Adobe Photoshop CS3 Extended version 10.0 software (Adobe Systems Inc., USA).

### 2.2. Phylogenetic Analysis

The large and small subunits of the nuclear ribosomal RNA genes (LSU, SSU) were included in the analysis. All sequences obtained from GenBank were used in Schoch et al. [8] and Zhang et al. [3] and are listed in Table 1. Sequences were aligned using Bioedit v7.2.0 version [9] and ClustalX v. 1.83 [10]. The alignments were checked visually and improved manually where necessary.

**Figure 1:**
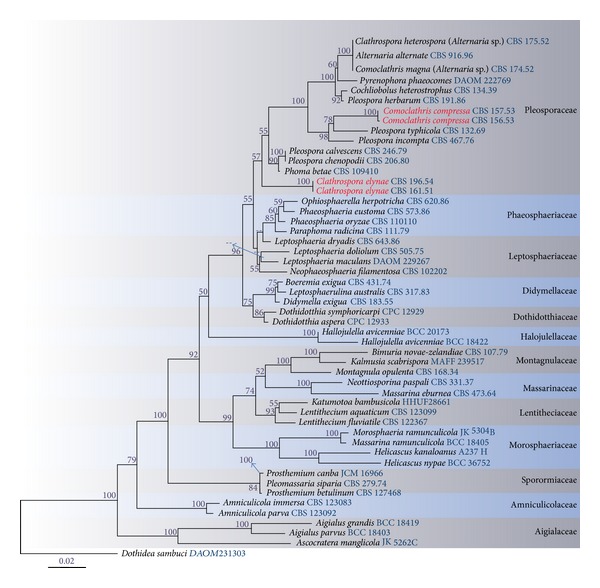
RAxML tree based on a combined dataset of SSU and LSU. Bootstrap support values >50% are shown above or below the branch. The putative strains of *Clathrospora elynae* (CBS 196.54 and CBS 161.51) and *Comoclathris compressa* (CBS 157.53 and CBS 156.53) are indicated in red. *Dothidea sambuci* is the out-group taxon. The original isolate numbers are noted after the species names.

Maximum Likelihood analysis was performed in RAxML [11] implemented in raxmlGUIv.0.9b2 [12]. The search strategy was set to rapid bootstrapping and the analysis was carried out using the GTRGAMMAI model of nucleotide substitution. The number of replicates was automatically inferred using the stopping criterion [13]. Maximum Likelihood bootstrap values equal or greater than 50% are given below or above each node (Figure 1). Phylogenetic trees were drawn using Treeview v. 1.6.6 [Page 2001].

## 3. Results

### 3.1. Molecular Phylogeny Based on Combined nrSSU and nrLSU

The combined 18 S and 28 S nrDNA data set comprised 52 taxa including strains of* Clathrospora elynae* (CBS 196.54 and CBS 161.51), *Comoclathris magna* (CBS 174.52), *Clathrospora heterospora* (CBS 175.52), and *Comoclathris compressa* (CBS 157.53 and CBS 156.53) with *Dothidea sambuci* as the out-group taxon. The 52 taxa analyzed in the cladogram formed 13 familial clades. Maximum Likelihood analysis used 1000 bootstrap replicates and yielded a tree with the likelihood value of ln:-9930.285726 and the following model parameters: alpha: 0.512987 and invar: 0.499567; Π(A): 0.259512, Π(C): 0.207265, Π(G): 0.277826, and Π(T): 0.255397. Phylogenetic trees obtained from maximum likelihood analyses yielded trees with similar overall topology at family and generic relationship in agreement with previous work [3, 4, 8].

### 3.2. Molecular Phylogeny of *Diademaceae*

Two putative strains of *Clathrospora elynae* (CBS 196.54 and CBS 161.51) which had been previously referred to *Diademaceae* by Lumbsch and Huhndorf [6] and Shoemaker and Babcock [1] were clustered in the family *Pleosporaceae *but separated from other genera of the family with a relatively high bootstrap value (55%). The type species of *Comoclathris*, *C. lanata*, was not available for study, but the two *Comoclathris compressa* strains cluster in a well supported clade within the *Pleosporaceae*, outside the *Alternaria* complex. Therefore we confer with Zhang et al. [4] and Woudenberg et al. [14] in transferring these two genera to *Pleosporaceae*. Two putative strains of *Comoclathris magna* (CBS 174.52) and *Clathrospora heterospora* (CBS 175.52) were clustered within the *Alternaria* complex as in Woudenberg et al. [14]. Woudenberg et al. [14] have tentatively considered *Comoclathris magna* (CBS 174.52) and *Clathrospora heterospora* (CBS 175.52) as *Alternaria* species. There is, however, confusion concerning the CBS 175.52 strain, because Dong et al. [15] used the name *Comoclathris baccata* in their paper for strain CBS 175.52 but submitted sequences to GenBank under the name *Clathrospora diplospora* [14]. In their study, Woudenberg et al. [14] have synonymised *Comoclathris baccata* with *C. heterospora*. We could not locate the type species of *Diadema*, *Diadema tetramerum*, and *Diademosa*, *Diademosa californiana*, for phylogenetic analysis due to the unavailability of sequence data. Therefore recollection, epitypification, and sequence data of *Diadema*, *Diadema tetramerum*, and *Diademosa*,* Diademosa californiana*, are necessary to validate *Diademaceae* genera and species relationships.

### 3.3. Taxonomy

*Diademaceae.* Shoemaker & C.E. Babc., Can. J. Bot. 70(8): 1618 (1992), MycoBank: MB 81955.

*Parasitic *or* saprobic* in stems and leaves. Sexual state: *Ascomata* subepidermal or subcuticular and later become superficial, globose, opening via flat circular lid, dark brown to black. *Peridium* thin, consisting of small pigmented thick-walled cells of *textura angularis*. *Hamathecium *of dense cellular pseudoparaphyses. *Asci* 8-spored, bitunicate, fissitunicate, clavate or ellipsoidal, short orbicular pedicel, without an ocular chamber. *Ascospores* partially overlapping to biseriate, fusiform, brown, with three or more transverse septa without longitudinal septa, mostly terete (cylindrical; frequently circular in section but narrowing to one end), mostly with a thick sheath. Asexual state: Unknown.

*Type: Diadema.* Shoemaker & C.E. Babc.

Shoemaker and Babcock [1] introduced *Diademaceae* which they considered to be a distinctive family comprising *Clathrospora*, *Comoclathris*, *Diadema*, *Diademosa*, and *Macrospora* whose species have ascomata opening by a flat circular lid [1]. The feature of ascomata opening via a flat circular lid was considered to be an adaptation to the alpine habitat [16]. Ascospores are fusiform, brown, with three or more transverse septa, with or without longitudinal septa, and frequently terete, usually with a thick sheath [1, 2, 4].

Lumbsch and Huhndorf [6] excluded *Macrospora *from *Diademaceae* and assigned it to *Pleosporaceae*, as it was considered to be a synonym for *Pyrenophora*. We have seen type material of *Macrospora scirpicola *and it is neither *diademaceous* nor *pleosporaceous *and therefore will be considered as subject of a future paper. Lumbsch and Huhndorf [6] also included *Graphyllium* in the family *Diademaceae*, but this classification has not been followed by many authors. Shoemaker and Babcock [1] and Zhang et al. [4] referred* Graphyllium* to the family *Hysteriaceae *based on its hysterothecium-like ascomata forming a longitudinal, slit-like opening. Shoemaker and Babcock [1] assigned *Clathrospora* to* Diademaceae* based on ascomata opening with an intraepidermal discoid lid and muriform applanate ascospores with more than one row of longitudinal septa. Ascomata, however, have slightly papillate ostioles and *Alternaria*-like asexual morphs, and recent molecular data shows that *Clathrospora *has an affinity with the family *Pleosporaceae *[4, 14]. *Platyspora* has been referred to *Diademaceae* [4] and was considered a synonym of *Graphyllium* [2, 4] or as a synonym of *Comoclathris* [5].

Species of the order *Pleosporales *with applanate ascospores were previously separated into three families (*Diademaceae*, *Hysteriaceae*, and *Pleosporaceae*) which differ in the way the ascomata open [2]. Ascomata openings by a flat circular lid were characteristic of the family *Diademaceae*, while species of *Hysteriaceae* open via a long narrow slit and species of *Pleosporaceae* open by a central pore [1]. Based on the above discussion we exclude* Clathrospora*,* Comoclathris*,* Diademosa*, and *Graphyllium* from the *Diademaceae*. Based on morphology and/or molecular data and at this time, we accept only *Diadema, *which has mostly terete ascospores (except *D. obtusa *which has flattened ascospores), in the family. *Diademaceae *is, however, not supported by molecular data, but no sequence data is available for the generic type *Diadema*. Further studies are required to resolve the phylogenetic relationship in the *Pleosporales*. In the light of all of the above, we retain the *Diademaceae *to include a single genus *Diadema *which has immersed, intraepidermal ascomata, opening via a flat circular lid, and asci with a short orbicular pedicel without an ocular chamber and ascospores are reddish-brown, usually cylindrical, and frequently circular in section but narrowing to one end with a distinct, mucilaginous sheath.

### 3.4. Accepted Genus in *Diademaceae*

*Diadema.* Shoemaker & C.E. Babc., Can. J. Bot. 67(5): 1349 (1989), MycoBank: MB 25293.

*Saprobic* on culms of grasses (*Poaceae*). *Sexual state*: *Ascomata* scattered, immersed, intra-epidermal, globose to subglobose, black to brown, smooth-walled and opening via a flat circular lid. *Peridium* 1-layered, composed of small pigmented thick walled compressed cells, base composed of small pigmented thick-walled cells of *textura angularis*. *Hamathecium *of dense, numerous, septate, hyaline, cellularpseudoparaphyses. *Asci *8-spored, numerous, bitunicate, fissitunicate, broadly-clavate, with a short orbicular pedicel, without an ocular chamber. *Ascospores* obliquely biseriate, broadly fusiform, usually cylindrical; frequently circular in section but narrowing to one end, brown to reddish-brown, without longitudinal septa, guttulate, smooth-walled or finely punctate, with wide, distinct mucilaginous sheath. *Asexual state*: Unknown.

**Figure 2:**
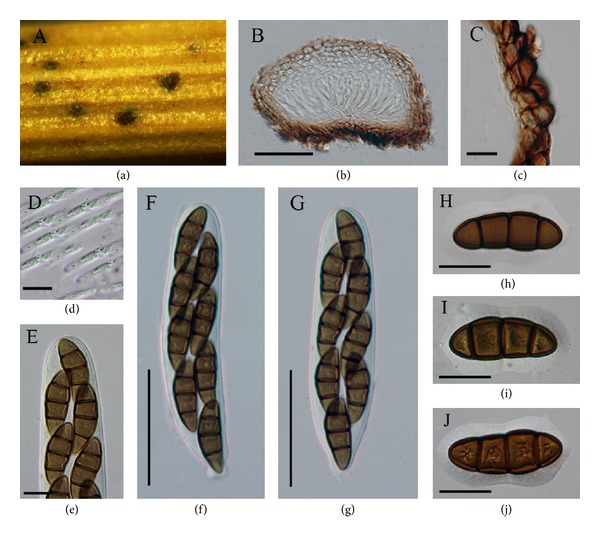
*Diadema tetramerum* (holotype). (a) Ascomata on substrate opening via a flat circular lid. (b) Vertical section of ascoma. (c) Closeup of the peridium. (d) Hyaline, septate pseudoparaphyses. (e) Apical part of the asci, ((f)-(g)) Asci with short orbicular pedicel. ((h)–(j)) Reddish-brown ascospores with broad sheath. Scale bars: (b) = 100 *μ*m, (c) = 10 *μ*m, ((d)–(g)) = 60 *μ*m, and ((h)–(j)) = 30 *μ*m.

*Type Species: Diadema tetramerum.* Shoemaker & C.E. Babc. [as “tetramera”], Can. J. Bot. 67(5): 1354 (1989), MycoBank: MB 136222 (see Figure 2).

*Saprobic* on culms of grasses (*Poaceae*). Sexual state: *Ascomata* 170–200 × 150–270 *μ*m ($\overset{-}{x}=190\times 250$ *μ*m, *n* = 10), scattered, immersed, intra-epidermal, globose to subglobose, black to brown, smooth-walled and opening via a flat circular lid. *Peridium* 10–22 *μ*m ($\overset{-}{x}=16$, *n* = 20), 1-layered, composed of small, pigmented, thick-walled, compressed cells, base composed of small, pigmented, thick-walled cells of *textura angularis*. *Hamathecium *of dense, 2-3 *μ*m diam ($\overset{-}{x}=2$, *n* = 20), numerous, septate, hyaline, cellularpseudoparaphyses. *Asci *100–150 × 20–25 *μ*m ($\overset{-}{x}=110\times 22$ *μ*m, *n* = 20), 8-spored, numerous, bitunicate, fissitunicate, broadly-clavate, with a short orbicular pedicel, rounded at apex without an ocular chamber. *Ascospores* 30–48 × 14–20 *μ*m ($\overset{-}{x}=44\times 13$ *μ*m, *n* = 40), obliquely biseriate, broadly fusiform, brown to reddish-brown, 3-transseptate, without longitudinal septa, guttulate, smooth-walled or finely punctate, with a distinct, 4-5 *μ*m wide, mucilaginous sheath. *Asexual state*: Unknown.

*Material Examined.* USA, California, Mt. Shasta, ridge south of Horse Camp, elevation 8250 ft, on culms of *Trisetum spicatum* (L.) Richter, 2 July 1947 W.B. Cooke 20223 (DAOM, holotype).

Shoemaker and Babcock [16] introduced *Diadema* and characterized the genus by large ascospores without longitudinal septa with a distinct mucilaginous sheath and ascomata with a circular lid-like opening. Currently eight species of *Diadema* are listed in Index Fungorum [5]. Six species were included when the genus was introduced and another two species (*Diadema ahmadii*, Kaz. Tanaka & S.H. Iqbal, and *Diadema sieversiae* (Peck) Huhndorf) were later added [4, 16]. The nature of the ascomata appears to be an important character of this genus and family. Except *D. obtusa* all other species of *Diadema* have terete; that is, ascospores are cylindrical, frequently circular in section but narrowing to one end. We observed *D. tetramerum*, the generic type of *Diadema* and besides ascomata opening via a circular lid, asci with the short orbicular pedicel without an ocular chamber and trans-septate, ascospores, lacking longitudinal septa, and surrounded by a very broad sheath narrowed to a waist near the middle septum are considered to be significant for the genus.

No molecular data is available for the type or other species of *Diadema*. Therefore recollection, epitypification, and sequence data is essential to establish family and species relationships.

### 3.5. Excluded Genera

*Clathrospora. *Rabenh., Hedwigia 1(18): 116 (1857).

*Saprobic *on wood and stems. *Sexual state*:* Ascomata* semi-immersed, scattered on putrid host stems and foliage, brown to blackish brown, subglobose or nearly globose, with a central sunken ostiole open via a circular lid, asci and pseudoparaphyses forming at the base of the peridium. *Peridium* composed of 3–5 layers of brown, relatively thick-walled cells of *textura angularis*, inner cells flattened, thin-walled and lighter. *Hamathecium *composed of dense, hyaline, filiform, pseudoparaphyses which are longer than the asci. *Asci* 8-spored, bitunicate, fissitunicate, thick-walled, cylindrical to clavate, with a short pedicle and shallow ocular chamber. *Ascospores* biseriate, fusiform 7-transseptate, two or many rows of longitudinal septa, muriform, constricted only at the central septum, dark brown to brown, surrounded by a thin, hyaline mucilaginous sheath. *Asexual State*:* Alternaria*-like.

**Figure 3:**
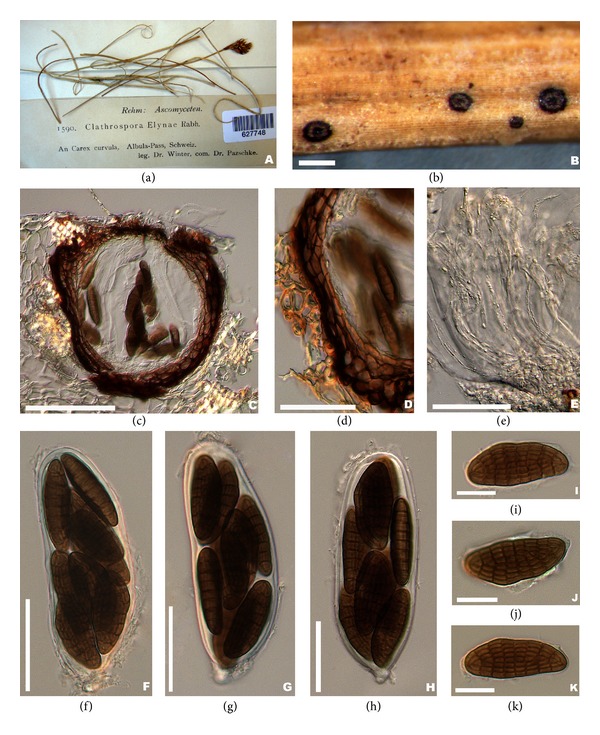
*Clathrospora elynae* (isotype). (a) Herbarium material. (b) Closeup of ascomata. (c) Section of the ascomata. (d) Closeup of the peridium (e) Hyaline, filiform, and pseudoparaphyses. ((f)–(h)) Cylindrical to clavate asci with a short pedicle and ocular chamber. ((i)–(k)) Dark brown to brown muriform ascospores surrounded by a thin, hyaline mucilaginous sheath. Scale bars: (b) = 100 *μ*m, (c) = 10 *μ*m, ((d)–(g)) = 60 *μ*m, and ((h)–(j)) = 30 *μ*m.

*Type Species: Clathrospora elynae.* Rabenh., Hedwigia 1 : 116 (1857) (see Figure 3).

*Saprobic *on wood and stems. *Sexual State*:* Ascomata* 140 × 220–145 × 175 *μ*m ($\overset{-}{x}=170\times 150$ *μ*m, *n* = 10), semi-immersed, scattered on the putrid host stems and foliage, subglobose or nearly globose, brown to blackish brown, with a central sunken ostiole open via a circular lid, asci and pseudoparaphyses forming on the base of the peridium. *Peridium* 20–55 *μ*m ($\overset{-}{x}=38$, *n* = 20), composed of 3–5 layers of brown, relatively thick-walled cells of *textura angularis*, inner cells flattened, thin-walled and lighter. *Hamathecium *composed of dense, 2-3 *μ*m diam ($\overset{-}{x}=2$, *n* = 20), hyaline, filiform, pseudoparaphyses, longer than the asci. *Asci* 160–230 × 24–48 *μ*m ($\overset{-}{x}=190\times 35$ *μ*m, *n* = 20), 8-spored, bitunicate, fissitunicate, thick-walled, cylindrical to clavate, with a short pedicle and ocular chamber. *Ascospores* 40–65 × 18–27 *μ*m ($\overset{-}{x}=53\times 23$ *μ*m, *n* = 40), biseriate, fusiform, 7-transseptate, two or many rows of longitudinal septa, muriform, constricted only at the central septum, dark brown to brown, surrounded by a thin, hyaline mucilaginous sheath.* Asexual State*:* Alternaria*-like.

*Material Examined.* Switzerland, on the stem of *Carex curvula*, September 1898, Winter (BPI 627748, isotype).

Shoemaker and Babcock [1] assigned *Clathrospora* to *Diademaceae* and included an additional nine species and provided a key to the genus based on the number of septa and length of ascospores.* Clathrospora* was characterized by circular lid-like opening and applanate, muriform ascospores. Currently, 50 *Clathrospora* species are listed in the genus in Index Fungorum [5]. Molecular studies based on combine gene analysis showed that two putative strains of *Clathrospora, C. elynae* (CBS 196.54) and *C. diplospora *(IMI 68086), were clustered in *Pleosporaceae *[4, 8]. We obtained similar results in the phylogenetic tree produced from combined nrLSU and nrSSU sequence analysis (Figure 1). *Clathrospora elynae* the type of *Clathrospora *formed a separate clade with relatively high bootstrap support (55%) within *Pleosporaceae*. Based on the phylogenetic result together with the morphological characters (slightly papillate ostiole and *Alternaria*-like asexual morph) we refer *Clathrospora* to *Pleosporaceae*. 

*Comoclathris.* Clem., Gen. fung. (Minneapolis): 37, 173 (1909) **≡*  Platyspora* Wehm., World Monograph of the Genus *Pleospora* and its Segregates: 254 (1961).

*Habitat* saprobic on dead wood or stems. *Sexual state*: *Ascomata* semi-immersed to superficial, scattered or aggregated, subglobose or nearly globose, brown to blackish brown coriaceous, ascomata opening via a large circular aperture or lid. *Peridium *comprising 3-4 layers of brown, relatively thick-walled cells of *textura angularis*. *Hamathecium* composed of dense, hyaline, filiform, septate pseudoparaphyses.* Asci* 8-spored, bitunicate, fissitunicate, cylindrical to cylindro-clavate, with an ocular chamber. *Ascospores* uniseriate or partially overlapping, fusiform, muriform, brown to reddish-brown, surrounded by a thick, hyaline, mucilaginous sheath. *Asexual State*:* Alternaria*-like

*Type Species: Comoclathris lanata.* Clem. [as “Comochlatris”], Gen. fung. (Minneapolis): 1–227 (1909). MycoBank: MB 209341.

*Comoclathris*, typified by *Comoclathris lanata*, was introduced by Clements (1909). The genus is characterized by ascomata with circular lid-like openings and applanate reddish-brown to dark reddish-brown, muriform ascospores, with single longitudinal septa [1]. Zhang et al. [4] tentatively placed *Comoclathris* in the *Pleosporaceae* based on *Alternaria*-like asexual morphs and this was followed by Woudenberg et al. [14]. *Comoclathris* shares common characters with *Pleospora herbarum,* the type of *Pleospora*, in having cylindrical to cylindroclavate asci with an ocular chamber and muriform, brown or pale brown, with or without sheath ascospores.* Comoclathris* and *Pleospora* differ in the opening of ascomata (opening via a large circular aperture or lid versus open by a central pore).* Comoclathris* and *Pleoseptum* share similar characters in having globose, black, ascomata, and cylindrical to cylindroclavate asci with muriform, yellowish to dark brown ascospores. *Comoclathris *differs from *Pleoseptum* in having superficial ascomata with circular lid-like openings composed of comparatively thin peridium and applanate and fusiform ascospores surrounded by a distinct hyaline, mucilaginous thick sheath [3, 4]. In *Pleoseptum *ascomata are immersed, usually with a papillate apex, with a relatively broad peridium and ovoid to fusoid ascospores [2, 3]. *Comoclathris *was considered to differ from *Clathrospora* as in the latter genus species have two or more rows of longitudinal septa as compared with a single row in *Comoclathris* [3]. Shoemaker and Babcock [1] provided a key to 21 species of *Comoclathris*. Presently 32 epithets are listed for *Comoclathris* in Index Fungorum [5]. Molecular data for *Comoclathris lanata*, the type species of *Comoclathris*, is not available. Two strains of *Comoclathris compressa* (CBS 157.53 and CBS 156.53), however, cluster together in a well-supported clade within the family *Pleosporaceae* [14]. Based on the phylogenetic result coupled with the morphological characters (*Alternaria*-like asexual morph) we agreed with Zhang et al. [4] and Woudenberg et al. [14] to place *Comoclathris* in *Pleosporaceae. *This is, however, based on a species and recollection of the type species is essential to establish the correct placement of the genus.

*Diademosa.* Shoemaker & C.E. Babc., Can. J. Bot. 70(8): 1641 (1992).

*Saprobic* on stems and wood. *Sexual state*: *Ascomata* immersed, initially erumpent becoming superficial, scattered, depressed-globose, some flattened at the base, opening a disc-like lid of brown prismatic cells with setae. *Peridium* composed of brown pseudoparenchyma cells of *textura angularis*. *Hamathecium *of numerous, dense, septate, hyaline, cellularpseudoparaphyses. *Asci* 8-spored, bitunicate, fissitunicate, clavate with short narrow pedicel and minute ocular chamber. *Ascospores *biseriate, partially overlapping, fusiform, straight, frequently circular in section but narrowing to one end, with transverse and vertical septa, pale brown to dark brown, smooth walled. *Asexual state*: Unknown.

**Figure 4:**
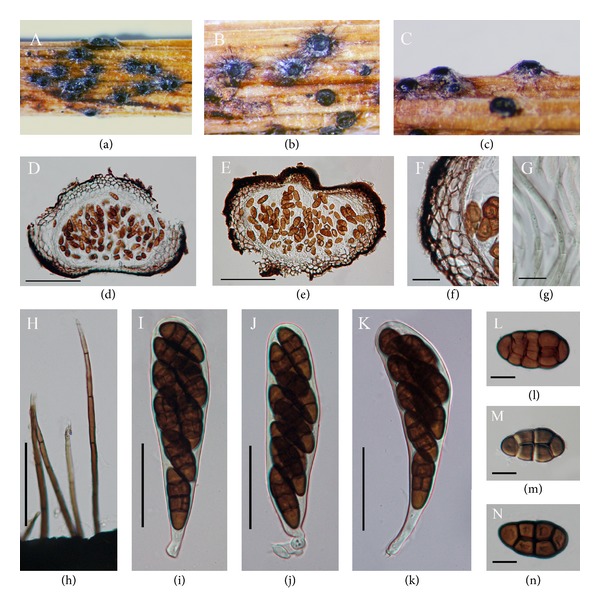
*Diademosa californiana* (holotype). ((a)-(b)) Ascomata on host substrate. (c) Side view of the ascomata. ((d)-(e)) Section of ascomata. (f) Section of peridium. (g) Septate, hyaline, and cellularpseudoparaphyses. (h) Light to dark brown seta. ((i)–(k)) Ascus with minute pedicel bearing irregularly arranged 8 ascospores. (l)–(n) Ascospores. Scale bars: ((d)-(e)) = 200 *μ*m, ((f)–(h)) = 10 *μ*m, ((i)–(k)) = 50 *μ*m, and (l)–(n) = 10 *μ*m.

*Type Species: Diademosa californiana.* (M.E. Barr) Shoemaker & C.E. Babc. [as “californianum”], Can. J. Bot. 70(8): 1641 (1992) *≡* *Graphyllium californianum* M.E. Barr, Mem. N. Y. bot. Gdn 62: 40 (1990) (see Figure 4).

*Saprobic* on stem and wood. *Sexual state*: *Ascomata* 200–365 × 240–425 *μ*m ($\overset{-}{x}=315\times 275$ *μ*m, *n* = 10), immersed, initially erumpent becoming superficial, scattered, depressed-globose, some flattened at the base, opening a disc-like lid of brown prismatic cells with setae. *Peridium* 24–59 *μ*m ($\overset{-}{x}=32$, *n* = 20), composed brown pseudoparenchyma cells of *textura angularis*. *Hamathecium *of dense, 2-3 *μ*m diam ($\overset{-}{x}=2$, *n* = 20), numerous, septate, hyaline, cellularpseudoparaphyses.* Asci* 140–175 × 24–28 *μ*m ($\overset{-}{x}=150\times 26$ *μ*m, *n* = 20), 8-spored, bitunicate, fissitunicate, clavate with short narrow pedicel and minute ocular chamber. *Ascospores *50–65 × 26–32 *μ*m ($\overset{-}{x}=57\times 29$ *μ*m, *n* = 40), biseriate or discontinuously arranged, partially overlapping, fusiform, straight, cylindrical; frequently circular in section but narrowing to one end, with transverse and vertical septa, muriform, constricted at first septum, pale brown to dark brown, smooth walled. *Asexual state*: Unknown.

*Material Examined.* USA, Bump-Cold Boiling Lake Trail, Lassen Volcanic National Park, Shasta, California, on branch of *Wyethia*, 12 July 1966, W.B. Cooke & D.L. Hawksworth. (NY, holotype).

*Diademosa* was established by Shoemaker and Babcock [1] and typified by *D. californiana*, based on the ascoma opening via a circular lid and ascospores being frequently circular in section, but narrowing to one end. *Diademosa californiana* was initially introduced as *Graphyllium californianum *by Barr [17] and referred to *Hysteriaceae *based on the pore or slit like opening. Reexamination of the type specimens by Shoemaker and Babcock [1] concluded that *Diademosa* opened by a flat lid similar to *Diadema* and assigned it into *Diademaceae*. The lid is hard to observe in sections unless they are mounted directly in lactic acid because excessive swelling occurs in water [1]. *Diademosa* differs from* Comoclathris* in having cylindrical, frequently circular in section, but narrowing to one end ascospores compared with flattened ascospores of *Comoclathris*. *Diademosa* and the generic type of *Pleosporaceae*, *Pleospora* share common characters. Both *Diademosa *and* Pleospora* comprise narrowly oblong ascomata with cellular pseudoparaphyses and cylindrical to clavate asci with muriform, brown or pale brown ascospores. However, *Diademosa* differs from *Pleospora* in having an ascomata opening via a circular lid, covered with setae and asci with short narrow pedicel, while *Pleospora* species have ascomata opening by a central pore without setae and asci with a short, thick, furcated pedicel. Except the ascomata opening via disc-like lid, *Diademosa *resembles some characters of *Pyrenophora*. That is, both *Diademosa* and *Pyrenophora* have superficial ascomata with setae and muriform, smooth-walled, light brown to dark brown ascospores. Currently four species of *Diademosa* are listed in Index Fungorum [5], but no molecular data is available for the genus. We place *Diademosa* in *Pleosporaceae* because of its similarities with other genera in this family, but confirmation of the phylogenetic status of this genus depends on recollecting the fungus and epitypification with molecular sequences. 

*Graphyllium.* Clem., Botanical Survey of Nebraska 5: 6 (1901).

*Habitat *saprobic on woody stems. *Sexual state*: *Ascomata *semi-immersed, hysteriform, black to brown, subglobose to ovoid. *Peridium* comprising 2-3 layers of brown, relatively thick cells of *textura angularis*, inner cells flattened, thin-walled and lighter.* Asci* 8 spored, bitunicate, fissitunicate, clavate. *Ascospores* biseritate overlapping, muriform, applanate, obpyriform, straight, with 3-4 transverse septa, 1–2 longitudinal septa or no longitudinal septa, brown to olive green. *Asexual state*: Unknown.

*Type Species*: *Graphyllium chloës.* Clem., Bot. Surv. Nebraska 5: 6 (1901) *≡* *Pleospora chloës* (Clem.) Petr., Sydowia 6(5-6): 337 (1952).

Initially *Graphyllium* was placed in the *Hypodermiaceae* by Clémencet (1901) and described as “Hysterothecium innate, then erumpent, linear, simple, membranaceous-plectenchymatous, black; asci ovoid or cylindrical-clavate, 8-spored; spores brown, elliptical to oblong, with transverse-and longitudinal septa, but not muriform; pseudoparaphyses simple or branched, septate, forming an epithecium." Later Barr [17] transferred the genus to order *Pleosporales* and referred to *Phaeosphaeriaceae*. *Platyspora* was considered as a synonym of *Graphyllium* [4]. Shoemaker and Babcock [1] assigned *Graphyllium *to *Hysteriaceae* considering the ascomatal characters along with applanate ascospores that are at least 3-septate in side view and have some longitudinal septa in front view. Lumbsch and Huhndorf [6] included *Graphyllium *in the family *Diademaceae*, but Zhang et al. [4] referred to *Hysteriaceae.* We examined the generic type of *Graphyllium*, *G. chloës* we also agreed to refer *Graphyllium* tentatively in *Hysteriaceae* because of its hysterothecium-like ascomata forming a longitudinal opening which is clearly deviated from the lid-like opening in *Diademaceae*. However the correct placement of this taxon still depends on epitypification with molecular data.

## 4. Concluding Remarks

The importance of molecular data in determining the importance of morphological characters and relationship of microfungi cannot be overstressed and has proved significant at establishing genus and species relationships [18, 19] and resolving cryptic species in important plant pathogenic genera, for example, *Diaporthe *[20] and *Pestalotiopsis* [21]. Shoemaker and Babcock [1] introduced *Diademaceae* which they considered to be a distinctive family based on the ascomata opening via a flat, circular lid and comprising *Clathrospora*, *Comoclathris*, *Diadema*, *Diademosa,* and *Macrospora* [1]. Recent studies based on molecular phylogeny [4, 14], including this study, conclude that *Clathrospora *and *Comoclathris* clustered within *Pleosporaceae*. Molecular data, however, is not available for* Diadema* and *Diademosa*. *Graphyllium* is placed in *Hysteriaceae* because of its hysterothecium-like ascomata with a slit like opening; this clearly diverges from the lid like opening in *Diademaceae*. The characteristic feature of ascomata opening via a flat circular lid is considered as an adaptation to the alpine habitat [16]. It is, however, doubtful if this character is significant and whether *Diademaceae* is a separate family in the order *Pleosporales*. Until further molecular data becomes available we maintain *Diademaceae* with a single genus *Diadema *based on its large transseptate ascospores surrounded by a distinct mucilage sheath and ascomata with a circular, lid-like opening [16].

## Figures and Tables

**Table 1:** Taxa used in the phylogenetic analysis and their corresponding GenBank numbers. Culture and voucher abbreviations are indicated where available.

Taxon	Culture	SSU	LSU
*Aigialus grandis *	JK 5244A	GU296131	GU301793
*Aigialus parvus *	BCC 18403	GU479743	GU479778
*Alternaria alternata*	CBS 916.96	KC584507	DQ678082
*Amniculicola immersa *	CBS 123083	GU456295	FJ795498
*Amniculicola parva *	CBS 123092	GU296134	FJ795497
*Ascocratera manglicola *	JK 5262C	GU296136	GU301799
*Bimuria novae-zelandiae *	CBS 107.79	AY016338	AY016356
*Boeremia exigua *	CBS 431.74	EU754084	EU754183
*Byssothecium circinans *	CBS 675.92	AY016339	AY016357
*Clathrospora elynae *	CBS 161.51	KC584628	KC584370
*Clathrospora elynae *	CBS 196.54	KC584629	KC584371
*Clathrospora heterospora *(*Alternaria* sp.)	CBS 175.52	KC584577	KC584320
*Cochliobolus heterostrophus *	CBS 134.39	AY544727	AY544645
*Comoclathris compressa *	CBS 156.53	KC584630	KC584372
*Comoclathris compressa *	CBS 157.53	KC584631	KC584373
*Comoclathris magna *(*Alternaria* sp.)	CBS 174.52	KC584578	DQ678068
*Didymella exigua *	CBS 183.55	EU754056	EU754155
*Dothidea sambuci *	DAOM 231303	AY544722	AY544681
*Dothidotthia aspera *	CPC 12933	EU673228	EU673276
*Dothidotthia symphoricarpi *	CPC 12929	EU673224	EU673273
*Halojulella avicenniae *	BCC 18422	GU371831	GU371823
*Halojulella avicenniae *	BCC 20173	GU371830	GU371822
*Helicascus nypae *	BCC 36752	GU479755	GU479789
*Katumotoa bambusicola *	MAFF 239641	AB524454	AB524595
*Lentithecium aquaticum *	CBS 123099	GU296156	GU301823
*Lentithecium fluviatile *	CBS 122367	GU296158	GU301825
*Leptosphaeria doliolum *	CBS 505.75	GU296159	GU301827
*Leptosphaeria dryadis *	CBS 643.86		GU301828
*Leptosphaeria maculans *	DAOM 229267	DQ470993	DQ470946
*Leptosphaerulina australis *	CBS 317.83	GU296160	GU301830
*Massarina eburnea *	CBS 473.64	GU296170	GU301840
*Montagnula opulenta *	CBS 168.34	AF164370	DQ678086
*Morosphaeria ramunculicola *	BCC 18405	GQ925839	GQ925854
*Morosphaeria ramunculicola *	JK 5304B	GU479760	GU479794
*Neophaeosphaeria filamentosa *	CBS 102202	GQ387516	GQ387577
*Neottiosporina paspali *	CBS 331.37	EU754073	EU754172
*Ophiosphaerella herpotricha *	CBS 240.31	DQ678010	DQ678062
*Phaeosphaeria eustoma *	CBS 573.86	DQ678011	DQ678063
*Phoma radicina *	CBS 111.79	EU754092	EU754191
*Pleomassaria siparia *	CBS 279.74	DQ678027	DQ678078
*Pleospora betae *	CBS 109410	EU754079	EU754178
*Pleospora calvescens *	CBS 246.79	EU754032	EU754131
*Pleospora chenopodii *	CBS 206.80	JF740095	JF740266
*Pleospora herbarum *	CBS 191.86	DQ247812	DQ247804
*Pleospora incompta *	CBS 467.76	GU238220	GU238087
*Pleospora typhicola *	CBS 132.69	JF740105	JF740325
*Preussia terricola *	DAOM 230091	AY544726	AY544686
*Prosthemium betulinum *	CBS 127468	AB553644	AB553754
*Prosthemium canba *	JCM 16966	AB553646	AB553760
*Pyrenophora phaeocomes *	DAOM 222769	DQ499595	DQ499596
*Sporormiella minima *	CBS 524.50	DQ678003	DQ678056
*Sporormiella minima *	CBS 524.50	DQ678003	DQ678056
